# New technologies to improve healthcare in low- and middle-income countries: Global Grand Challenges satellite event, Oxford University Clinical Research Unit, Ho Chi Minh City, 17th-18th September 2019

**DOI:** 10.12688/wellcomeopenres.16008.2

**Published:** 2020-08-13

**Authors:** Minh Ngoc Dinh, Joseph Nygate, Van Hoang Minh Tu, C. Louise Thwaites, Global Grand Challenges Event Vietnam Group

**Affiliations:** 1 School of Science & Technology, Royal Melbourne Institute of Technology University, Ho Chi Minh City, Vietnam; 2 Oxford University Clinical Research Unit, Ho Chi Minh City, Vietnam; 3 Centre for Tropical Medicine and Global Health, University of Oxford, Oxford, UK

**Keywords:** LMIC, healthcare, machine learning, artificial intelligence, technology, engineering

## Abstract

We report the outputs of a satellite event in Ho Chi Minh City, Vietnam, organized as part of the “2 ^nd^ Global Grand Challenges of Engineering Summit”. The event considered challenges and potential solutions for improving low- and middle-income country (LMIC) healthcare systems, with particular reference to critical care.  Participants from key regional and local stakeholders in healthcare and engineering discussed how new advances in technology, especially in the field of Artificial Intelligence, could be of potential benefit. This article summarizes the perspectives and conclusions of a group of key stakeholders from LMICs across South and South East Asia.

## Introduction

In September 2019, The UK, US and Chinese academies of engineering co-hosted the 2nd Global Grand Challenges Summit in London. This event, inspired by the ‘14 Grand Challenges of Engineering’ involved engineers, researchers, innovators, entrepreneurs, and policymakers from around the world to discuss the theme ‘Engineering in an Unpredictable World’. As part of the summit, satellite events were held in India, Kenya, Mexico, Thailand, Uganda and Vietnam to discuss globally relevant topics related to the principle theme. In this report, we summarize the outputs of the Vietnamese event, which brought together key regional and local stakeholders in healthcare and engineering to discuss challenges and potential benefits of introducing new technologies to improve healthcare in low- and middle-income countries (LMICs).

### Care quality in low- and middle-income country healthcare systems

Healthcare systems in many LMICs have undoubtedly improved over the last few decades. Areas such as maternal health and preventative medicine have benefited from a sustained drive to implement universal standards of care. Nevertheless, a recent report by the Lancet Global Health Commission estimated that almost 9 million lives and $1.6 trillion in productivity are lost each year as a result of poor quality medical care, the majority of which occurs in LMICs ^1^. Important limitations in diagnosis and treatment were identified as causes of this, in addition to systems-level problems with safety, integration and continuity of care. Overall quality of care was worst in vulnerable groups, such as the low-income groups, and those with stigmatized conditions ^1^.

The Lancet Commission argues that providing any health system that is not of high quality is unethical ^2^. However, in improving care quality, health systems face many challenges, particularly with regard to critical illness, where providing healthcare is most complex, requiring highly-trained staff and expensive equipment for diagnostics and treatment. These challenges are often common to all resource settings, however in LMICs where resources are already limited, overcoming them may be more difficult.

This satellite event focused on the provision of high-quality care to critically ill patients and enabled a wide variety of engineering and healthcare stakeholders from the region to share perspectives on the potential for new technologies to improve health care and particularly critical care in LMIC settings.

## Challenges to providing high quality care of critically ill patients: perspectives from South and Southeast Asian LMICs

### Access to care

In many LMICs, there is wide variation in access to healthcare services, and particularly large differences between care available to urban and rural communities. Throughout the world, in critically ill patients, when rapid assessment and treatment are necessary, ensuring timely access to services for remote communities is a particular challenge. In high-income settings, dedicated retrieval services have been employed to transfer critically ill patients to specialized centres. These are expensive and rely on non-specialist medical staff to triage and stabilize patients. In LMICs, even if there are rural health stations, staff may often have little or no medical training at all, and there are even fewer options to safely transfer their patients.

### Appropriate diagnosis and treatment

Timely identification of critical illness and prompt implementation of treatment are vital in improving outcome in seriously ill individuals. Indeed, delayed diagnosis and slow initiation of treatment were both identified as the main reasons for poor quality of care by the Lancet Care Quality Commission ^2^. However, there are important contextual differences between LMICs and high-income settings, which necessitate innovative solutions to these challenges. For example, the causes of critical illness themselves are often different. In low-income countries, more than half of all deaths are due to maternal causes, nutritional deficiencies or communicable diseases compared to just 7% in high-income settings ^3^. This means diagnosis may \ require different laboratory infrastructure and equipment in LMICs. In almost all critical illness, once a diagnosis has been reached, treatment requires expensive equipment and careful monitoring to assess response to treatment and anticipate complications early. Whilst these may be available in LMICs, usually this is only in a limited number of specialist centres ^4^.

### Health systems

LMIC health systems vary widely between countries making quality improvement measures and benchmarks difficult to compare. Increasingly, private providers provide critical illness care, but standards are variable, and lack of comprehensive regulatory systems are a further challenge to implementation of high-quality care. Corruption within some healthcare systems has been cited as a major barrier to advancement and sustainability of quality care , taking forms such as favouritism, informal payments, absenteeism or data manipulation ^5^. An estimated 10–25% of global health spending is lost to corruption with unquantifiable impact on lives, communicable disease control or antimicrobial resistance ^5^. In most healthcare systems, about 70% of recurrent healthcare resources are spent on people. In many LMICs, there are particular deficiencies in numbers and distribution of appropriately trained staff, thus improving management, distribution and training can have a huge impact on healthcare quality and outcomes ^6^. As lack of knowledge amongst healthcare providers has been identified as a factor in itself preventing further development, WHO have stated that improving training and knowledge should be a priority ^6^.

### Cost of care

Critical care is costly due to the expensive treatments, sophisticated equipment and labour-intensive care required. Although healthcare coverage is increasing, in LMICs, many of these expenses are still passed directly as out-of-pocket costs to patients and their families ^7^. Currently about 100 million people are pushed into extreme poverty every year as a result of out-of-pocket medical costs ^8^. Additionally, many survivors are left with long-term disability which, in addition to costs of hospitalization, puts huge economic strain on families and communities.

Until now, intensive care units (ICUs) in LMICs have adopted similar models of care to those used in high income settings. However, the associated requirement for staff, equipment and training is limiting if not prohibitive in most LMICs ( Table 1). Recent advances in engineering and technology may negate this need for costly staff and equipment, offering disruptive and novel alternatives to conventional care approaches.

**Table 1.** Barriers to providing high quality ICU care as identified by event participants.

Access	Limited number of critical care beds. Often highly centralized and difficult to access from remote areas
Cost	Lack of universal healthcare coverage High out-of-pocket costs to families and patients
Staff: numbers, training, accessibility	Low numbers of staff Less highly-trained staff Better trained staff concentrated in a few urban centres
Equipment and infrastructure	Lack of equipment (expensive) Difficulty maintain equipment Harsh operational environments (heat, humidity, power cuts etc) Lack of supportive infrastructure (imaging, laboratory etc)
Health systems	Lack of community services Lack of health system integration Limited health system data available

## Recent advances in engineering and technology in the healthcare context

### Artificial Intelligence and Machine Learning: definitions and applications in healthcare

**Figure 1.**
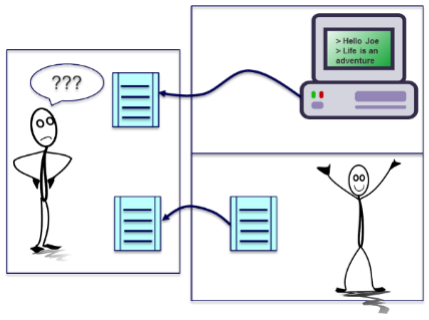
The Turing Test.

"Artificial Intelligence" (AI) refers to a field of computer science that accentuates the creation of intelligent machines that operate and react like humans. However, the general goal of AI is not well-defined because there is no general consensus on what specifically constitutes intelligence. Alan Turing, via his famous Turing Test, defined the goal of AI is to produce responses that are indistinguishable from those of a human ( Figure 1) ^9^. Early applications of AI in healthcare included expert systems, such as MYCIN ^10^, which assisted physicians in diagnosing blood infections, and DENDRAL ^11^, which aided chemists in determining the structure of organic molecules. Unfortunately, these expert systems, which relied on static sets of predefined rules, failed to address the dynamic and the probabilistic nature of medical phenomenon and human activities.

**Figure 2.**
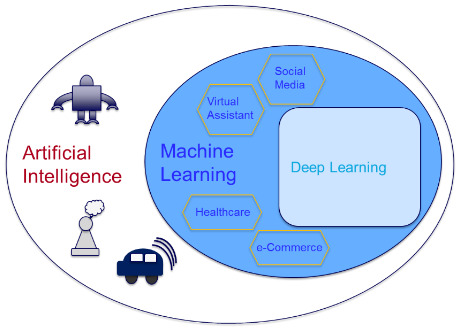
From Artificial Intelligence to Deep Learning.

Recently, Machine Learning (ML) and Deep Learning (DL) have gained more attention as principled frameworks to implement AI in the age of Big Data ^12, 13^. ML focuses more on improving the learning and the adaptation capability of machines and computer systems, given the continuing changes in its operational context, while DL introduces the neural network-based methodology where the learning process loosely emulates the information processing and distributed communication nodes in biological systems. Figure 2 puts AI, ML and DL into perspective, in which ML is a subfield of AI and DL is a specific methodology to improve the machine’s capacity to learn. ML is seeing gradual acceptance in the healthcare industry thanks to the capacity to analyze large sets of medical data in order to provide timely risk scores, precise resource allocation, and illness diagnosis. We review some major applications of AI and ML in improving the state-of-the-art in healthcare.

***Enhanced diagnosis.*** WHO estimate that up to two third’s of the world’s population lack access to chest X-rays and diagnostics ^14^. Whilst in many LMIC ICU settings, chest X-rays are available, there are limited number of experts able to interpret these images. AI, and DL in particular may provide a potential solution to this problem as they are well-suited to pattern recognition Increasing numbers of publications show DL applications to chest radiography where a wealth of high volume datasets allow algorithms to be constructed ^15^. Whilst to date, there are few examples specific to ICU or critical care, algorithms are able to distinguish common ICU-related X-ray findings, for example Chexnet, a deep learning algorithm detected pneumonia better than radiologists with up to 25 years of experience ^16^. Whilst most studies pertain from high income settings, a recent study demonstrates that a DNN used to analyse chest X-rays from Indian hospitals perform similarly to four experienced radiologists ^17^. AI has also been applied to MRI or CT. For example, DL systems improved accuracy of lung cancer detection from low-dose CT and a ML system for MR breast cancer detection has received FDA approval ^18– 20^. Many LMIC ICUs lack access to CT and MRI, thus currently AI systems related to X-ray interpretation are potentially of greatest value. Here the ability to detect changes over time and with often sub-optimal images is a particular challenge to AI systems. AI and ML methods can also be applied to other modalities to aid diagnosis. With particular interest to critical care are AI methods of interpreting vital sign waveforms. Algorithms applied to arterial blood pressure waveforms have been used to predict intra-operative hypotension and similarly to intracranial pressure waveforms predict the onset if raised intracranial pressure ^21, 22^. Whilst these are clearly of utility in high income settings, in LMICs with little access to invasive monitoring, such systems may be of less value. 

Reproduced under a Creative Commons Attribution License, which permits unrestricted use, distribution, and reproduction in any medium, provided the original author and source are credited.

**Figure 3.**
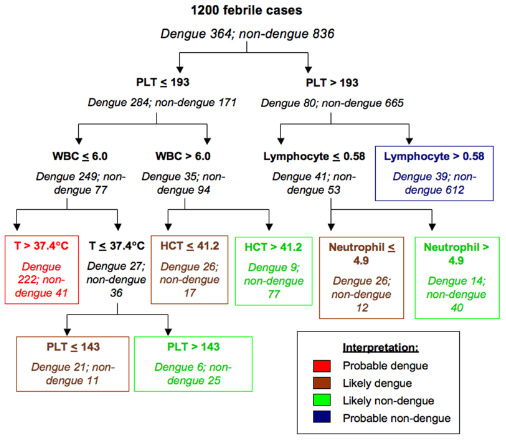
A decision tree for dengue diagnostics ^27^.

***Decision support.*** ML offers a framework for analysis of high-dimensional multimodal data, which is of particular advantage in examining complex biomedical data, and shows promise in improving detection, diagnosis, and monitoring of disease. For critical care where there are often huge volumes of data, ML approaches are particularly attractive. In high income settings ML systems for early warning scores prognosis or sepsis prediction have been developed ^23– 26^. Examples include recurrent neural networks to provide real-time prediction of post-cardiosurgical complications such as mortality, renal failure, and postoperative bleeding ^26^, and prediction of optimal treatment sepsis using reinforcement learning ^24^. In this latter example, reinforcement learning methods were employed on a large multimodal critical care dataset to identify optimal treatment strategies for patients in sepsis. These were then tested using a different dataset, showing that patients who received treatments closest to those suggested by the AI algorithm had improved outcome. There are limited data from LMICs and it is not clear whether HIC algorithms can be applied, especially as monitoring data in LMIC ICUs is much more limited in both frequency and type. Tanner *et al*. ^27^ develop decision tree algorithms that are capable of separating dengue from other febrile illnesses in the primary care setting ( Figure 3).

***Healthcare systems.*** To aid healthcare management, ML applications can be developed to better identify and track chronic disease states and high-risk patients, design appropriate interventions, and reduce the number of hospital (re)admissions and claims. For example, BERG’s Interrogative Biology platform uses ML to identify the molecular basis of efficacy and adverse events in order to map disease and treatments in oncology, neurology and other rare conditions ^28^. Such technology allows healthcare providers to take a more predictive approach rather than relying on trial-and-error. With growing use of electronic healthcare records in LMICs, these technologies are increasingly relevant to resource-limited health systems, particularly as many systems are designed around costing and billing. As already discussed, costs of ICU in many in LMICs results in huge out-of-pocket expenses. Better understanding of costs of ICU care may be able to allow appropriate interventions, use of resources and protect these vulnerable populations against excessive or disproportionate costs.

***AI healthcare potential for critical care in LMICs.*** The above mentioned AI systems have potential for significant impact in LMICs and address many of the barriers to providing high quality ICU care as identified by the event participants. Reducing the cost and expertise needed to monitor and treat critically ill patients is an important step not only in improving patient outcomes *per se*, but also in reducing inequalities in service provision. For example, the requirement for highly trained radiology staff can be reduced with DL systems. Busy and less well-trained staff can be supported by ML clinical decision support systems trained or optimized on relevant contextualized data. Furthermore, as more countries embrace electronic health records, data from these could be used either for clinical decision support or healthcare service optimization.

There are already examples from event participants of initiatives towards using these technologies in LMICs. In Vietnam, ML-based clinical decision support tools for tetanus and dengue are being developed as well as DL image-analysis in tuberculous meningitis and dengue as part of the VITAL (Vietnam ICU Technology Applications Laboratory) project.

Nevertheless, despite these potential advantages there remain several challenges and limitations to the adoption of AI technologies.

### Other emerging technologies in healthcare

A new generation of information technologies including internet of things (loTs), big data, cloud computing, and crowdsourcing, has transformed healthcare to become not only more efficient and more convenient, but also more personalized, yet deliverable at low-costs. For example, patients can be equipped with wearable devices to monitor their health constantly. Another example is that of low-cost mobile devices can be used as live source of data for monitoring spread of diseases. We identify several trends in which healthcare systems, and in particular critical care, in LMICs can benefit.

***Smart healthcare.*** The smart healthcare model focuses on enabling real-time monitoring and immediate feedback of health data in order to deliver timely medical interventions. This model drives on the emergence of implantable/wearable devices, and smart health information platforms, which are connected by IoT technology. In particular, by integrating advanced sensors with high-performance microprocessors, wearable/implantable devices can continuously sense and monitor various physiological indicators of patients in an intelligent manner. Another system developed by RMIT researchers detects human respiration using WiFi devices ^29^. The system does not require subjects to wear a device at all. Such technologies are particularly attractive in LMIC settings where wearable devices and monitoring systems (e.g. commodity WiFi devices) may be much cheaper (often <10% cost) and allow remote monitoring. Thus, solutions like this can support clinical care for isolated communities, requiring little equipment or significant on-site medical expertise. The primary challenges for such systems are the limited battery life and maintaining a wireless network connection. Nevertheless, these technologies have shown to be improving comfort, while allowing sensed data to be combined with health information for better and more timely medical intervention.

 There are many other uses beyond the ICU or for patients undergoing post-ICU rehabilitation. For example, the HCMC University of Technology and Education, Vietnam, demonstrated IoTs-based fall detection system, in which data collected from tri-axial accelerometer sensors and/or Kinect camera systems are transferred continuously to a cloud server for processing and detecting fall states ^30^. Fall detection and alerts can be sent to relatives or healthcare personnel for immediate medical assistance. Data could similarly be used to monitor recovery and rehabilitation as post-discharge medical services rarely exist in LMICs.

**Figure 4.**
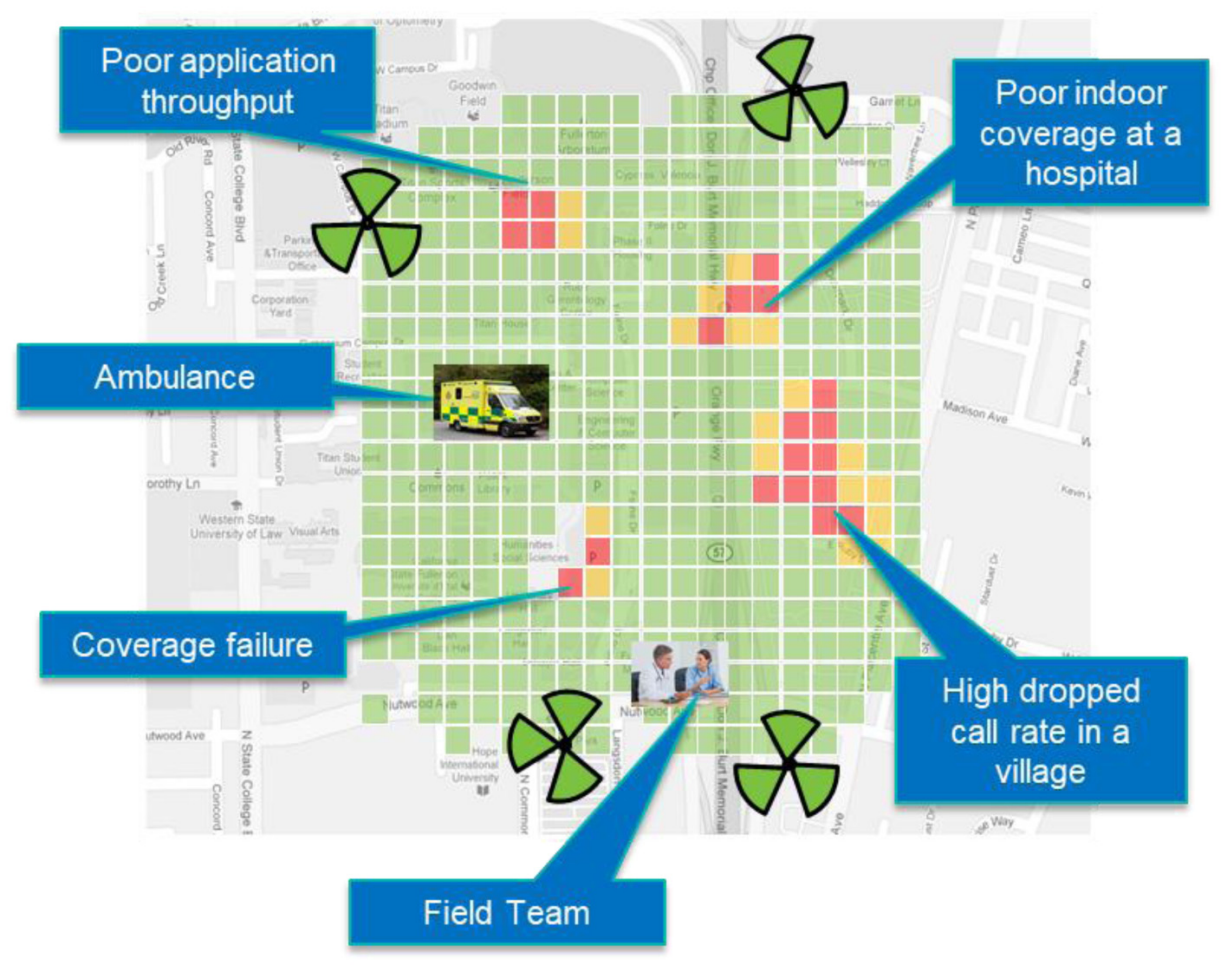
Monitoring emergency responders’ locations. Figure adapted from "Applying Machine Learning in Managing Deployable Systems," 2018 IEEE International Symposium on Technologies for Homeland Security (HST) ^31^.

***Crowdsourcing and Big Data.*** The concept of Crowdsourcing is to utilize the vast wealth of the public data to address social challenges including healthcare. For example, collecting and analyzing geolocation data from sensor-based and mobiles devices allows monitoring the spread of diseases or levels of air pollution. Such capacity provides data to better understand causes of disease or can enable prevention and control. Other uses of crowdsourcing data with geolocation technologies include measuring and predicting network performance and coverage, monitoring emergency responders’ locations, tracking and backtracking disease carriers, and determining the effectiveness of quarantine and isolation ( Figure 4).

In the critical care setting, large amounts of data are already routinely collected. In high income countries, national-level datasets are routinely gathered and are an invaluable resource for improving care quality and patient outcomes ^32^. Improving the quality of these data in LMICs would facilitate similar improvements in these countries. One example of a successful platform is in Sri Lanka where an ICU registry provides accurate real-time data for network partners using a cloud-based platform ^33^. This platform has been expanded and adopted by 9 countries as part of the CRIT CARE Asia network and adopted in over 44 sites across the region ^34^. Data from the registry allows quality improvement initiatives and audit, with demonstrable benefits in ICU patient outcomes ^4^.

**Figure 5.**
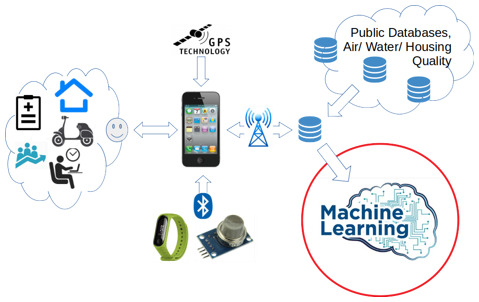
mHealth infrastructure to support online monitoring of patient’s condition.

***mHealth and telemedicine.*** To date, smartphone ownership worldwide surpasses three billion and continues to grow in the next few years. In 2018, 48% of the global population were connected to the internet, and in LMICs mobile phones were the primary medium for this ^35^. South and Southeast Asia notably have amongst the world’s most affordable mobile internet making these countries ideal sites for telemedicine services. In Vietnam 40% of the population are expected to have a smartphone by 2021. Such uptakes introduce the opportunity for mHealth, which focuses on improving the quality, efficiency and cost of healthcare via mobile platforms (see Figure 5). For example, a Cloud Telemedicine Information system, which consists of 100 devices to measure blood pressures and heart rate, can obtain live patient data to enable physicians to monitor patient’s blood pressures online ^36^. This pilot cyber medical system, developed by the School of Biomedical Engineering of International University - Vietnam National Universities in Ho Chi Minh City, was successfully implemented in Binh Duong province (Vietnam) to test its efficacy. At the University of Medicine and Pharmacy Ho Chi Minh City, the Department of Family Medicine leads a project connecting family doctors and patients through telemedicine. Whilst currently these projects mainly focus on non-acute care, there is potential for similar technologies to be used to support ICU care in remote sites, or for patients after discharge from hospital. The main focus of telemedicine in acute ICU care would be support of clinicians remotely. Such initiatives are being explored in Vietnam to support clinicians caring for patients with tetanus outside of specialist centres.

### Issues of adopting emerging technologies in healthcare

Despite much interest and enthusiasm in the technologies described above, the application in patient care has some limitations. Compared to traditional statistical analysis tools, many AI solutions (particularly DL) are considered ‘black boxes’ because outputs from AI models lack transparency and their rationale cannot be clearly explained. Using systems without clear biologically-plausible reasoning concerns many clinicians and regulators, especially if results have direct impact on patient care. There are critical questions around ethics, such as who is responsible for biases produced by AI. Finally, some practitioners consider AI a ‘hype’ because its recent success in other disciplines mainly due to the advent of brute-force computing power and the availability of more data. This sentiment generates caution in adopting AI and ML solutions in patient care and clinical practices. For mHealth and Big Data technologies there are concerns about data privacy and ownership. These issues may be particularly pertinent in LMIC settings where regulation and control may be lacking ( Table 2). Additionally, concepts of data privacy and sharing are often very different in LMICs in our region and principals applied from HIC are not always acceptable. Establishing clear regulations in this area is, however a priority to allow appropriate development and application of these technologies.

**Table 2.** Barriers to adoption of Artificial Intelligence (AI) in low- and middle-income country healthcare settings.

Poor quality of data or insufficient volume due to data, especially heath care data, is often in inconsistent formats and consists of a lot of noise and bias; limited infrastructure to collect data
Integration into existing clinical workflows
Lack of skilled staff to lead and use AI because it is challenging to find and employ staffs with both healthcare background and Machine Learning skills
No clear benefits from using AI because medical doctors often found AI outputs lack transparency to support medical decisions
Regulatory and legal requirements

## Summarys and next steps

Improving the provision and quality of critical care in South and Southeast Asia is a significant step towards achieving sustainable development goals and improving quality of life in the region. Heterogeneity of health systems, remote rural populations and cost of providing critical care are significant barriers to achieving this.

During the satellite event in Vietnam, we identified a range of technology advances that are beneficial to healthcare systems in LMICs. However, these may have significant disruptive potential to conventional models of care provision, but ultimately offers cost-effective solutions for LMICs in the region. Nevertheless, significant barriers exist before such technologies can be widely employed, including technical, regulatory and behavioural challenges. This multidisciplinary meeting enabled professionals from relevant backgrounds to discuss key elements of this. Attendees made a firm commitment to maintaining working together in the future. This includes activities such as an international meeting in 2020, shared student projects and new research initiatives.

## Disclaimer

The views expressed in this article are those of the author(s). Publication in Wellcome Open Research does not imply endorsement by Wellcome.

## Data availability

No data is associated with this article.
