# Supplementary material for: New technologies to improve healthcare in low- and middle-income countries: Global Grand Challenges satellite event, Oxford University Clinical Research Unit, Ho Chi Minh City, 17th-18th September 2019
Source: Wellcome Open Res. 2020 Aug 13;5:142. Originally published 2020 Jun 15. [Version 2] doi: 10.12688/wellcomeopenres.16008.2 (PMC7443781; doi:10.12688/wellcomeopenres.16008.2)
Supplement: Peer review history

# Peer review: Reviewer response for version 2

**DOI:** 10.21956/wellcomeopenres.17778.r39970

**Published:** 2020-08-21

**Authors:** James Malycha

**Affiliations:** 1 Nuffield Department of Clinical Neurosciences, University of Oxford, Oxford, UK

This revision is excellent and addresses all the issues raised. There are some very small typo's so just do a final edit before final indexing.

Does the article adequately reference differing views and opinions?

Partly

Are all factual statements correct, and are statements and arguments made adequately supported by citations?

Yes

Is the Open Letter written in accessible language?

Partly

Where applicable, are recommendations and next steps explained clearly for others to follow?

Partly

Is the rationale for the Open Letter provided in sufficient detail?

Partly

Reviewer Expertise:

The deteriorating ward patient

I confirm that I have read this submission and believe that I have an appropriate level of expertise to confirm that it is of an acceptable scientific standard.

# Peer review: Reviewer response for version 2

**DOI:** 10.21956/wellcomeopenres.17778.r39969

**Published:** 2020-08-13

**Authors:** Alejandra Aranceta Garza

**Affiliations:** 1 Department of Biomedical Engineering, University of Strathclyde, Glasgow, UK

Authors have clarified all my comments.

Does the article adequately reference differing views and opinions?

Yes

Are all factual statements correct, and are statements and arguments made adequately supported by citations?

Yes

Is the Open Letter written in accessible language?

Yes

Where applicable, are recommendations and next steps explained clearly for others to follow?

Yes

Is the rationale for the Open Letter provided in sufficient detail?

Yes

Reviewer Expertise:

Development of medical technology.

I confirm that I have read this submission and believe that I have an appropriate level of expertise to confirm that it is of an acceptable scientific standard.

# Peer review: Reviewer response for version 1

**DOI:** 10.21956/wellcomeopenres.17558.r39107

**Published:** 2020-06-30

**Authors:** James Malycha

**Affiliations:** 1 Nuffield Department of Clinical Neurosciences, University of Oxford, Oxford, UK

**Reason for conference: focus on the provision of high-quality care to critically ill patients, enable various interested parties to provide input on potential for new technologies to improve health care (critical care specifically).**

**Challenges:**

**Access to care:**

- *Rapid assessment and treatment are necessary*
  In most developed countries, roughly 30% of critical care involves the management of non-acute patients requiring peri-operative care or patients with multiple comorbidities who are at higher risk of complications than other cohorts. I am unsure of the percentage in LMIC but it is probably reasonable to note that not all critical care patients are those that require rapid assessment and management plans.
- *…for remote communities, it is a particular challenge*
  This is also the case for developed countries, and we get around this issue by funding expensive and skill dense retrieval services. The point being that I don’t think this is an issue that is unique to LMIC. What may be worth commenting on is that these countries (may?) have a higher proportion of their populations in rural settings, making the logistics of servicing the population more onerous.  A retrieval logistics are very expensive!
- *Even if there are rural health stations, staff may often have limited medical training and few options to safely transfer their patients to larger centres*
  Retrieval services are very expensive (as mentioned above) and they have a very specific process of acquiring and maintaining skills (the details of which I am not knowledgeable). In Australia, the process of stabilising a critical patient in a remote location is often left to a non-critical care trained person until the retrieval service arrives. This is a hypothesis, but for this small aspect of critical care service provision perhaps LMIC countries are not as challenged as in other aspects?

**Appropriate diagnosis and treatment:**

- *In low-income countries, more than half of all deaths are due to maternal causes, nutritional deficiencies or communicable diseases compared to just 7% in high-income settings. This means diagnosis may often require more highly developed laboratory infrastructure and equipment in LMICs.*
  I am concerned about this statement. I don’t understand how maternal, nutritional and communicable disease critical care presentations require more developed lab infrastructure than, for example, trauma or cardiothoracic presentations which are very prevalent in developed countries? I’m not sure how this is distinctive for LMIC. They probably require different lab infrastructure. It may be worth emphasising this point. Or it may be worth emphasising that lab infrastructure is not as widely available (if that is the case, which I assume it might be).
  Likewise, diagnostic tests being time-consuming doesn’t seem a key issue in LMIC where the cost and availability of labour may be less of an issue than it is in developed countries. I don’t think that is a major barrier to good care being provided. It certainly requires further expertise. Overall, my best guess (having had no experience in such a setting I must emphasise this it is a guess) is that a significant challenge is the provision and maintenance of highly trained clinicians and laboratory scientists/technicians working in concert in a timely manner for critical unwell patients. That is expensive both materially and in terms of human capital and is difficult to achieve when the social structures within which hospitals exist are not sufficiently developed. This involves extremely complex manufacturing, engineering and educational supply chains (about which I know very little) but may warrant mentioning and/or exploring. I'd certainly be interested to learn more.

**Health systems:**

- *Private health care provision is growing in LMIC, which is unregulated and standards vary. Additionally, they are prone to corruption (paraphrased). *
  That seems very important but a broad description of the health systems as they currently exist in both settings and setting out a clear and concise description of the main differences and why that matter clinically in the critical care setting. A table would be intuitive and would help the reader. And there is no description of how/when/why corruption is more damaging to health systems in LMIC than in developed countries. I’d be very interested to know more.
- *Recognising the need to invest in the development, training and knowledge of clinical staff (paraphrased).*
  This seems logical and important, but this statement is true of all health systems, developed or otherwise. How are their distinctions in the LMIC setting? How can these be targeted? With specific discussion around emerging technologies?

**Costs of care:**

- *Currently about 100 million people are pushed into extreme poverty every year as a result of out-of pocket medical costs. Additionally, many survivors are left with long-term disability which, in addition to costs of hospitalization, puts huge economic strain on families and communities…*
  This statement is startling and is of huge importance. One hypothesis might be that private health care provision (as mentioned above) is filling the gaps left by struggling publicly funded health systems – which is possibly a ‘double edged sword’? I.e. because these private health systems are financially motivated, insufficient consideration and regulation is being adopted to prevent serious financial hardship post critical care. This raises important ethical/moral questions that might be useful to touch on briefly. It would seem logical to think such families in need of critical care support (which is usually life threatening) are vulnerable to predatory providers whose primary motivation is making money. How can we quantify this? Use technology to defend against this? Perhaps this opens an interesting line of thought around financial/tax data being used to indirectly improve critical care provision in LMIC? I.e. Maybe we need to think outside the box a bit?  
- *…Recent advances in engineering and technology, however, offer disruptive and novel alternatives to conventional care approaches.*
  This is encouraging but the author has provided no further detail on the matter. What disruptive and novel alternatives? Having been informed on the above issue, this would be useful to know. Little of what follows in the paragraphs below explores that in great detail.

**Recent advances in engineering/tech in Healthcare:**

**AI/ML definitions:**

- The descriptions of AI/ML are slightly confusing. These paragraphs are lacking a clear focus.

 

**Enhanced Diagnosis:**

- The author makes the point that AI/DL are particularly well adapted to pattern matching in radiology (although the 2 examples are for cancer detection, which has little or no relevance to most critical care imaging requirements). They then go on to mention that this might be helpful in terms of negating fatigue. That does seem useful but wouldn’t a major challenge in LMIC be accessing highly trained medical staff? As such, a more obvious advantage might be the use of AI systems that quickly and reliably interpret radiological images in place of a doctor/under the remote supervision of a doctor (if indeed that AI can function at a similar level of accuracy). There are many caveats to that statement because the interpretation of an image also requires an understanding of the clinical context. This is particularly true in critical care, where the rapid evolution of pathologies need to be regularly reviewed, and forward and backward comparisons made to enable decision making. I would also guess that remote radiology might be highly applicable to LMIC where the availability of expertise might be limited? Figure 1 is also somewhat confusing.

**Decision support:**

- As above, these two examples have little or no relevance to helping decision making in critical care. The example provided (Komorowski) is highly relevant and it might be helpful to expand on exactly what they did, which was extremely interesting, innovative and exciting. It was slightly limited in certain ways and expanding on the positives and negatives of this example might be useful.

**Healthcare systems:**

- As above, examples not highly relevant to critical care. There are many applications of ML being used for the deteriorating patient (Churpek in particular). It might be worth mentioning those.

**AI healthcare potential for critical care in LMICs:**

*The above-mentioned AI systems have potential for significant impact in LMICs and address many of the barriers to providing high quality ICU care as identified by the event participants*

 In light of what has been mentioned above, this statement needs to be better reinforced. I don’t think the examples mentioned above demonstrably show potential to address the barriers to providing good critical care. Although I concede this might be open to interpretation. Regardless, I would also add that those barriers (described in the first section) have not been described sufficiently clearly and that would make a nice addition to the paper. Perhaps a table? E.g. Barriers to the provision of critical care in LMIC:

1. It’s very expensive – how expensive? What is the comparison? Where might savings be made? What are the potential advantages of LMIC?
2. It’s reliant on complex supply chains? Perhaps describe one or two as examples? Like a blood gas machine, a CT machine and an Infectious Diseases lab?
3. It’s reliant on highly trained personnel – what are the barriers here?
4. Retrieval is challenging.
5. Public Health Service provision is limited.
6. Private Health Service provision is patchy and ethically ambiguous – this seems very important. Are there data to further inform this point in the paper?

**Other emerging technologies:**

**Smart healthcare:**

- *Timely intervention of medical behaviour*
  This sentence is confusing. Perhaps try it another way.
  Wearables are still in the early stages but are evolving fast. Perhaps some critical care relevant examples in this paragraph. There is considerable work being done in Oxford on this. You mention trends where LMIC might benefit. Please describe them.
- *Such technologies are particularly attractive in LMIC critical care settings where wearable monitoring systems may be much cheaper*.
  Can you provide examples of the cost comparison? This would be informative and relevant.
- *….and even allow remote monitoring and clinical decision tools to support patient care in isolated communities.*
  This is an interesting point. The logistics of such an exercise would be very interesting to learn about. Perhaps consider expanding on this briefly.
  You then expand on non-critical care related research – this is not relevant to the remit of this paper.

**Crows sourcing and Big Data:**

- Good points.

**mHealth and telemedicine:**

- Good points. However, critical care medicine is a particularly ‘hands on’ specialty making it difficult to extract benefit from telemedicine without careful planning. Was there anything in the conference discussion around this point?

**Issues of adapting emerging tech into health care: **

- You have mentioned: 1. Black box issues 2. Lack of clinician ‘buy in’ 3. Ethics 4. Data privacy – each of these seem to have merit but my best guess is that these four issues are not the key rate-limiting-steps when it comes to adapting tech into the LMIC critical care setting.
- I hypothesise the following are at least as or more important: ongoing technical limitations, the real difficulties around merging data-driven algorithmic outputs with health care systems that are (essentially) run by people, data inaccuracy, data delay, software/hardware maintenance, lack of data science expertise. I hasten to add I am not an expert in the LMIC domain so these are just guesses.

**Conclusions:**

- The barriers you mention were not really mentioned above. I think the conclusion should be a brief synopsis of the above points followed by your subjective interpretation of what this means.

**Summary statement by the Reviewer:**

- Thanks for the opportunity to review this interesting and important paper. It is an important topic that requires the attention and resources of the international critical care community. I have made suggestions which may help the author but I hasten to add I am not an expert on LMIC critical care provision so please interpret these comments with that in mind. There are some grammatical issues that need to be addressed. The headings are helpful but I’d argue that the examples provided are often not relevant to critical care, and those that are not sufficiently explored. The overall format of the paper might be worth simplifying and the author may consider adding in a table or two to summarise the key points. It also gives the impression that the discussions were very broad but not specific. This seems reasonable, but it might be helpful to mention that in the paper.

Does the article adequately reference differing views and opinions?

Partly

Are all factual statements correct, and are statements and arguments made adequately supported by citations?

Yes

Is the Open Letter written in accessible language?

Partly

Where applicable, are recommendations and next steps explained clearly for others to follow?

Partly

Is the rationale for the Open Letter provided in sufficient detail?

Partly

Reviewer Expertise:

The deteriorating ward patient

I confirm that I have read this submission and believe that I have an appropriate level of expertise to confirm that it is of an acceptable scientific standard, however I have significant reservations, as outlined above.

# Response

**Published:** 2020-07-23

**Authors:** C Louise Thwaites

**Affiliations:** Oxford University Clinical Research Unit, Vietnam

The article is meant to reflect the proceedings of an event focused around LMICs rather than provide a comprehensive review of the field. Nevertheless, the comments have allowed us to include a high-income setting perspective into the article and many of the challenges faced by critical care services in LMICs are also present in resource-rich settings. We have reflected this in the second version of our article. Answering Specific points:

 1. The reviewer raises an important point about the use of ICU beds for routine surgery or planned admissions. The participants however mainly wanted to discuss critically ill patients, not necessarily in ICU. We have clarified this by changing the sentence from critical illness to ‘critically ill patients when rapid access and treatment are necessary’.

 2. Ensuring access to care in rural communities is challenging in all communities and the text has been modified. However we feel in resource restricted settings challenges are more pronounced. Regarding rural health stations, our meaning here is that in LMICs, many rural health stations are staffed by non-health professionals with no or limited *medical *(certainly not critical care training) and transfers are done by foot, taxi or at best ambulances, usually accompanied by relatives only. Our participants felt that these were indeed different to HIC services.

 3. Our section about differences in diagnosis was not a clear and the reviewer correctly queries this. The statement and has been revised in line with the comment and our original meaning. The group was trying to convey that the necessary infrastructure was different.

 4. The section regarding private healthcare and corruption was also not clearly written and we have rephrased it to convey our meaning, i.e. that health care systems are variable and therefore difficult to compare and that the increased use of private healthcare makes it difficult to institute or evaluate quality improvement. Again the reviewer has correctly noted that this is not necessarily different in LMICs. The section on corruption has been expanded, but the article by Garcia (citation number 5) provides an excellent summary of the impact of corruption from the perspective of somebody directly involved in LMIC health service provision.

 5. Concerning staff and training, these of course are problems in all healthcare settings, but our group felt that the difference in LMICs is that there are less resources to start with, therefore less staff, and that these staff are also much less equally distributed. This has been clarified in the text and readers can refer further to the WHO report specifically on this issue (citation number 6).

 6. In many LMICs, private health providers are actively encouraged to develop to increase health system capacity. I am not aware of countries looking at the issues of financial hardship and many will rely on insurance companies to regulate cost of services. Generally private hospitals cater for more wealthy people with health insurance and it is not the private hospitals which are pushing people into poverty, sometimes the very poor will use them for convenience and speed. The approach in most countries is to aim for universal healthcare coverage. It is an interesting point that data may be useful to improve care and many countries are implementing electronic healthcare records which is very often driven by the need to provide medical insurance companies with accurate data. We have expanded the section on ‘big data’ to include this as a possibility.

 7. We have expanded the sentence on potential roles of new technologies as an introduction to the latter sections.

 8. The introductory paragraph on AI and ML have been altered. The first paragraph discusses AI as a field of study. The following paragraphs are on the application of AI in healthcare.

 9. The enhanced diagnosis has been modified to include more ICU examples, but also note their limitations regarding LMIC application at present.

 10. The decision support section has been expanded and further critical care examples included. Limitations regarding LMIC application are added.

 11. Health systems in this section and our event, aimed to look at the larger scale health systems (mainly hospitals). Examples of critical care systems have been included in the above section. The value of ML applied to electronic health records and resource allocation/ costs of ICU care has been added.

 12. A table of barriers to critical care provision has been added.

 13. Examples of smart healthcare systems have been expanded, although as this section is meant to be more broad and less focused on critical care this has been reflected in the examples. As there are many different examples, an exact cost comparison is not possible but an approximate percentage estimate has been added.

 14. We have expanded on the potential application of remote monitoring. We leave the more broad examples of local applications in this document but included why the examples, for example the fall-monitoring systems, are still relevant to ICU populations.

 15. The section on telemedicine in ICU has been expanded and clarified with ICU examples added.

 16. Regarding barriers to AI, this was not meant to be a comprehensive list but reflected views of participants.  We have included infrastructure in the table. The other points are covered already in the table.

 17. The conclusion represents the conclusion to the meeting. We have therefore expanded and re-titled this section to address the comments and be more clear.

# Peer review: Reviewer response for version 1

**DOI:** 10.21956/wellcomeopenres.17558.r39105

**Published:** 2020-06-15

**Authors:** Alejandra Aranceta Garza

**Affiliations:** 1 Department of Biomedical Engineering, University of Strathclyde, Glasgow, UK

This open letter touches on very important issues in trying to improve healthcare provision and adoption of new technologies in LMIC. It reflects on an international event hosted at the Oxford University Clinical Research Unit in Ho Chi Minh City, Vietnam.

 There are many important aspects that they discuss, from the complex (and almost impossible) task of a technology developed for HIC to be adopted by LMICs. The authors explained the different barriers and challenges in providing high-quality care in South and Southeast Asia, which is a complex, multivariate problem.

 This letter comments on the importance and relevance of using machine learning and deep learning algorithms in order to help provide the best quality of care for LMICs, whilst not posing an excessive burden on an already tired healthcare system. 

 I believe, there are a couple of aspects that could be improved:

1. Figure 1 is a bit confusing, maybe the authors can add detail to Figure captions.
2. The authors could invite for further research/action by providing a list of recommendations from the satellite event.
3. The complexity of data protection in adopting cloud data systems was not discussed, this is a crucial aspect of telehealth and should be addressed from the beginning. If there was no discussion around the topic at the satellite event, the authors could perhaps make a note of it for future research on the topic. 

Does the article adequately reference differing views and opinions?

Yes

Are all factual statements correct, and are statements and arguments made adequately supported by citations?

Yes

Is the Open Letter written in accessible language?

Yes

Where applicable, are recommendations and next steps explained clearly for others to follow?

Yes

Is the rationale for the Open Letter provided in sufficient detail?

Yes

Reviewer Expertise:

Development of medical technology.

I confirm that I have read this submission and believe that I have an appropriate level of expertise to confirm that it is of an acceptable scientific standard.

# Response

**Published:** 2020-07-23

**Authors:** C Louise Thwaites

**Affiliations:** Oxford University Clinical Research Unit, Vietnam

We have clarified Figure 1 to make it less confusing.

 Unfortunately we did not as a group make any recommendations for future research. This would be a valuable component of any future meeting and is an excellent suggestion.

 We have expanded the section about data security/ ethics in line with the comment.
